# Trio Exome Sequencing in VACTERL Association

**DOI:** 10.1016/j.ekir.2024.12.006

**Published:** 2024-12-09

**Authors:** Jasmina Ćomić, Erik Tilch, Korbinian M. Riedhammer, Melanie Brugger, Theresa Brunet, Katharina Eyring, Katharina Vill, Silke Redler, Velibor Tasic, Eberhard Schmiedeke, Frank-Mattias Schäfer, Nora Abazi-Emini, Ekkehart Jenetzky, Nicole Schwarzer, Anke Widenmann, Martin Lacher, Michael Zech, Sabine Grasshoff-Derr, Michaela Geßner, Carmen Kabs, Barbara Seitz, Andreas C. Heydweiller, Oliver Muensterer, Bärbel Lange-Sperandio, Udo Rolle, Johannes Schumacher, Matthias C. Braunisch, Riccardo Berutti, Heiko Reutter, Julia Hoefele

**Affiliations:** 1 Institute of Human Genetics, Klinikum rechts der Isar, Technical University of Munich, TUM School of Medicine and Health, Munich, Germany; 2 Department of Nephrology, Klinikum rechts der Isar, Technical University of Munich, TUM School of Medicine and Health, Munich, Germany; 3 Division of Nephrology, Boston Children's Hospital, Harvard Medical School, Boston, Massachusetts, USA; 4 Department of Pediatric Neurology and Developmental Medicine and LMU Center for Children with Medical Complexity, Dr. von Hauner Children's Hospital, Ludwig-Maximilians University, Munich, Germany; 5 Institute of Human Genetics, Medical Faculty and University Hospital Düsseldorf, Heinrich-Heine University, Düsseldorf, Germany; 6 University Children’s Hospital, Medical Faculty of Skopje, North Macedonia; 7 Clinic for Paediatric Surgery and Paediatric Urology, Klinikum Bremen-Mitte, Bremen, Germany; 8 Department of Pediatric Surgery and Urology, Cnopf'sche Kinderklinik, Nürnberg, Germany; 9 Institute of Integrative Medicine, Witten/Herdecke University, Herdecke, Germany; 10 Department of Pediatric and Adolescent Psychiatry and Psychotherapy, University Medical Centre, Johannes Gutenberg University of Mainz, Mainz, Germany; 11 SoMA, The German Patient Support Organization for Anorectal Malformations and Hirschsprung Disease, Munich, Germany; 12 Patient Organisation for Esophageal Diseases KEKS e.V., Stuttgart, Germany; 13 Department of Pediatric Surgery, University of Leipzig, Leipzig, Germany; 14 Institute of Neurogenomics, Helmholtz Munich, Neuherberg, Germany; 15 Institute of Advanced Study, Technical University of Munich, Garching, Germany; 16 Pediatric Surgery Unit, Buergerhospital and Clementine Kinderhospital, Frankfurt, Germany; 17 KfH-Board of Trustees for Dialysis and Kidney Transplantation (KfH-Kuratorium für Dialyse und Nierentransplantatione.V.), Munich, Germany; 18 Department of Paediatrics Surgery, Muenchen KlinikgGmbH, Munich Clinic Schwabing, Munich, Germany; 19 Department of General, Visceral, Vascular and Thoracic Surgery, Unit of Pediatric Surgery, University Hospital Bonn, Bonn, Germany; 20 Department of Pediatric Surgery, Dr. von Hauner Children's Hospital, Ludwig-Maximilians University, Munich, Germany; 21 Division of Pediatric Nephrology, Department of Pediatrics, Dr. v. Hauner Children's Hospital, Ludwig-Maximilians University, Munich, Germany; 22 Department of Pediatric Surgery and Pediatric Urology, University Hospital Frankfurt/M., Frankfurt am Main, Germany; 23 Institute of Human Genetics, University Hospital of Marburg, Marburg, Germany; 24 Department of Neonatology and Pediatric Intensive Care, University Erlangen-Nürnberg, Erlangen, Germany; 25 Institute of Human Genetics, University Hospital, Ludwig-Maximilians University, Munich, Germany

**Keywords:** burden test, exome sequencing, mitochondriopathy, VACTERL association, VACTERL-like phenotype, *ZNF417*

## Abstract

**Introduction:**

Currently, there is only limited data on monogenic causes of vertebral defects, anorectal malformations, cardiac defects, esophageal atresia or tracheoesophageal fistula, renal malformations, and limb defects (VACTERL) association. The aim of this study was to extend the spectrum of disease-causing variants in known genes, to determine the diagnostic yield of monogenic causes, and to identify candidate genes and rare variants by applying comprehensive genetic testing or rare variant burden.

**Methods:**

The total cohort comprised 101 affected individuals and their parents. Trio exome sequencing was only performed in 96 individuals and their parents because of DNA quality reasons and case-control gene and pathway burden tests were calculated and evaluated by quantile-quantile plots, principal component analysis plots and family-based association test (FBAT).

**Results:**

In 5 of 96 individuals, disease-causing variants in known genes or loci were identified to be associated with the following 4 disorders: Kabuki syndrome, Sotos syndrome, MELAS syndrome, and deletion syndrome encompassing *TWIST1*. In 91 individuals, no disease-causing variants were found. FBAT showed 14 significant variants, 2 significant genes (*LOC645752* and *ZNF417*), and 8 significant pathways.

**Conclusion:**

This study shows that most individuals with VACTERL association do not have known discrete genetic syndromes, implying that pathomechanisms or variants not identifiable by exome sequencing may exist requiring further investigation.

## Graphical abstract

**Figure:**
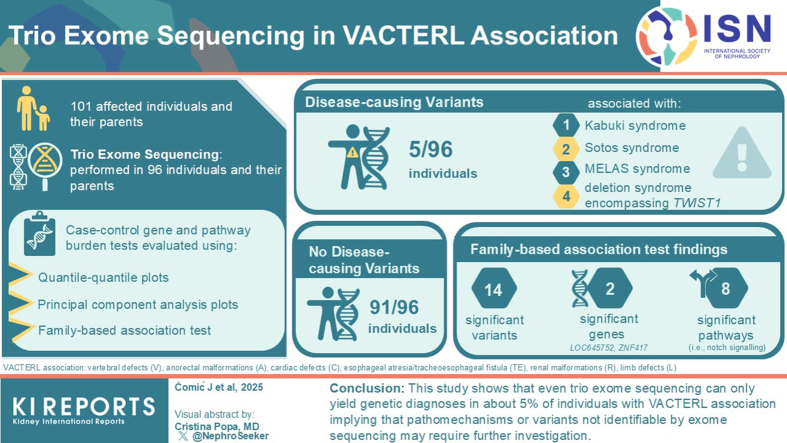

The acronym VACTERL is an etiologically and clinically heterogeneous disease that includes a combination of the following congenital malformations: V, vertebral; A, anorectal; C, cardiac; TE, trachea esophageal; R, renal and L, limb. The frequency of vertebral anomalies in these individuals has been estimated to range from 60% to 80%, anorectal malformations (ARMs) from 55% to 90%, cardiac anomalies from 40% to 80%, trachea esophageal with or without esophageal atresia from 50% to 80%, renal anomalies from 50% to 80% and limb malformations from 40% to 50%. Although there is still no firm consensus regarding the diagnostic criteria, most clinicians and researchers consider a minimum of 3 of the above-mentioned malformations to be present to diagnose VACTERL. Individuals with 2 component features have been termed VACTERL-like.^1^^,^^2^ Approximately 90% of the cases appear to be sporadic.^2^ Although little is known about the cause of VACTERL or VACTERL association X-linked with or without hydrocephalus, there is evidence that, in addition to the few known monogenic causes of this phenotypic association (e.g., *ZIC3*, *TRAP1,* or mitochondrial genes), a number of environmental factors (e.g., pregestational diabetes mellitus, assisted reproductive techniques, maternal pregestational overweight and obesity, and maternal smoking) are involved in the development of such malformations.^1^^,^^3^^,^^4^ In addition, disease-causing variants in genes such as *B9D1*, *FANCA,* or *FREM1* associated with Meckel syndrome, Fanconi anemia, or bifid nose with or without anorectal and renal anomalies, can mimic a VACTERL association.^3^

The estimated frequency of VACTERL association, which ranges from 1 in 10,000 to 1 in 40,000 newborns, could be attributed to the varying diagnostic criteria applied across different studies conducted both in the pregenomic and genomic eras, making accurate estimation challenging.^1^^,^^2^ In addition, infants currently born with VACTERL association have a much better prognosis than decades ago, because of the advantages of modern medicine.^2^ Using targeted animal models, multiple signaling pathways have been identified, suggesting potential (likely) pathogenic variants in the Sonic Hedgehog pathway, involving for example, Sonic Hedgehog, *GLI2*, and *GLI3* genes; nicotinamide adenine dinucleotide (NAD) pathway; and p53 signaling pathway.^5^, ^6^, ^7^ These pathways lead to a broad range of developmental abnormalities in mutant mice that reflect features of VACTERL association.^7^ Shi et al. identified variants in the *HAAO* and *KYNU* genes that inhibit the *de novo* synthesis of NAD and cause several congenital malformations similar to those seen in VACTERL association. Of note, the NAD *de novo* synthesis pathway metabolizes tryptophan to produce NAD. Niacin supplementation during pregnancy in mice prevents the development of embryonic malformations caused by NAD deficiency. Furthermore, disruption of pathways involving Hox and retinoic acid signaling may contribute to the pathogenesis.^2^^,^^8^^,^^9^

Molecular and cytogenetic testing using karyotyping and microarray analysis are standard techniques to search for genetic causes in these individuals.^10^ Larger studies using comprehensive molecular genetic testing, such as exome sequencing in this context, are rare. The most promising way to gain insights into VACTERL development is the identification of biological pathways and their underlying genetic alterations. Therefore, the aim of this study was the identification of disease-causing variants in disease-associated target genes, as well as of rare variants in candidate genes identified by exome sequencing, and by performing a gene and pathway burden test on the largest VACTERL or VACTERL-like cohort so far.

## Methods

### Study Population

**Figure 1:**
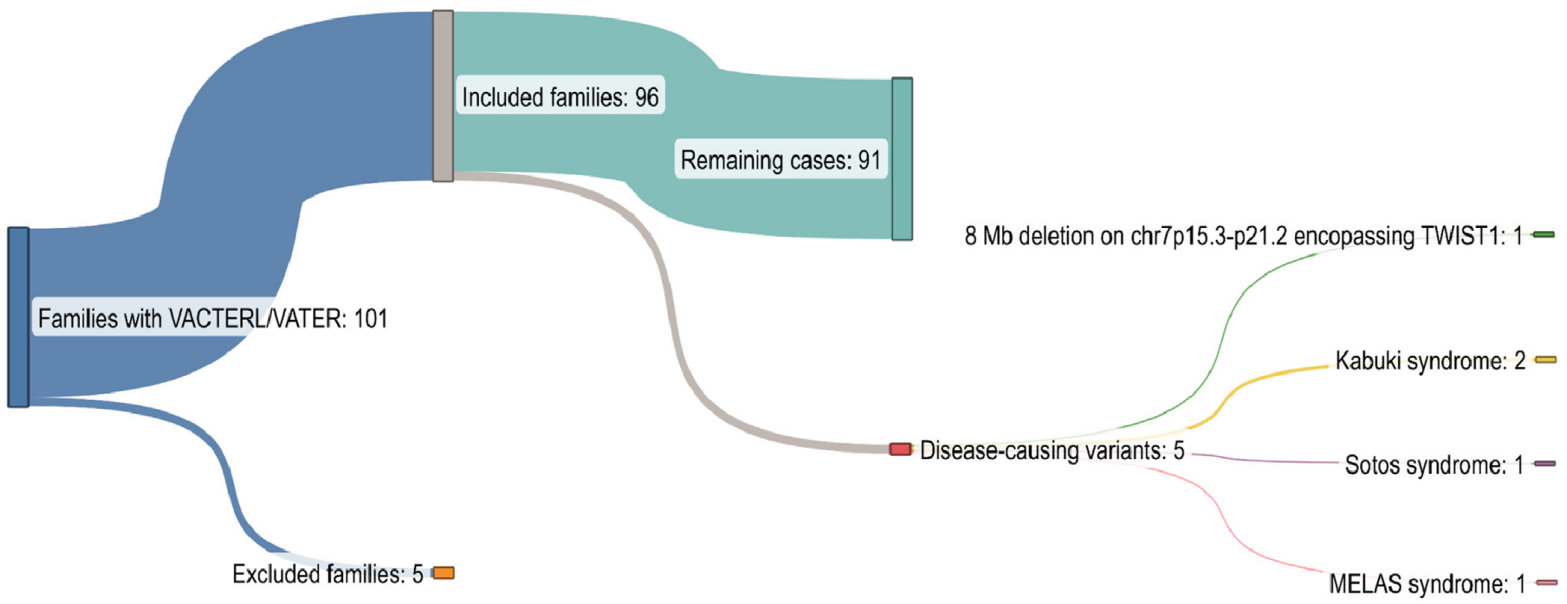
Flow chart of the study cohort. This figure was created with the free web-based tool SankeyMATIC (http://sankeymatic.com/build/). VACTERL/VATER, (V) vertebral defects, (A) anorectal malformations (ARM), (C) cardiac defect, (TE) tracheoesophageal fistula with or without esophageal atresia, (R) renal malformations, (L) limb defects.

Unrelated index cases of VACTERL association with their unaffected parents were recruited into the participating centers for this study. Clinical and phenotypic data were obtained from clinical reports and medical history, using a standardized questionnaire. Index cases were categorized in 1 of the following 2 subgroups according to the individual’s phenotype: (i) VACTERL group and (ii) VACTERL-like group (Figure 1). The individuals were collected from May 2016 to November 2022.

Affected individuals and families were recruited in collaboration with the participating children’s hospitals. Additional support was provided by the German self-help organizations for individuals with congenital ARM (SoMA e.V.) and tracheoesophageal malformations (KEKS e.V.). Before recruitment, the network partners of CURE-Net (German network for congenital urorectal malformations) and associated network partners generated standardized case report forms that were used in this study.

### Exome Sequencing

Exome sequencing for all affected individuals and their healthy parents was performed using Sure Select Human All Exon V5 (50Mb) Kit (Agilent Technologies, Inc., Santa Clara, CA) and a HiSeq2500 (Illumina, Inc., San Diego, CA), or with Sure Select Human All Exon V6 (60 Mb) Kit (Agilent Technologies, Inc., Santa Clara, CA) and a HiSeq4000 (Illumina, Inc., San Diego, CA).^11^ Mitochondrial DNA was derived from off-target exome reads as previously described.^12^ Reads were aligned to the Genome Reference Consortium Human Build 37 (UCSC Genome Browser build hg19) using Burrows-Wheeler Aligner (v.0.7.5a). SAMtools (version 0.1.19) was used for detection of single-nucleotide variants and small insertions and deletions. ExomeDepth was used to detect copy number variations (CNVs). A noise threshold of 2.5 was accepted for diagnostic analysis.^13^ The retrieved CNVs were visualized using Integrative Genomics Viewer (https://software.broadinstitute.org/software/igv/) to verify that the regions examined were adequately covered and that the CNVs were plausible. CNVs were then compared with publicly available control databases such as the Genome Aggregation Database (https://gnomad.broadinstitute.org/about), the Database of Genomic Variants (http://dgv.tcag.ca/dgv/app/home), and databases for CNVs such as DECIPHER (https://decipher.sanger.ac.uk/) and ClinVar (https://www.ncbi.nlm.nih.gov/clinvar/).

### Variant Interpretation

For the analysis of heterozygous (*de novo* or autosomal dominant inheritance) and mitochondrial variants, only variants with a minor allele frequency of <0.1% in the in-house database containing >27,100 exomes were considered. For the analysis of homozygous, compound heterozygous, or hemizygous, variants (autosomal recessive and X-linked inheritance), only variants with a minor allele frequency of <1.0% and a single-nucleotide variant quality of 30 were considered.^14^^,^^15^ Variants were checked in publicly available databases for (likely) pathogenic variants. These databases were ClinVar (https://www.ncbi.nlm.nih.gov/clinvar/), the Human Gene Mutation Database (HGMD Professional, http://www.hgmd.cf.ac.uk), and the Leiden Open Variation Database (https://www.lovd.nl). The variants were rated in accordance with the guidelines of the American College of Medical Genetics and Genomics and current amendments.^16^, ^17^, ^18^, ^19^

### Prioritization of Candidate Genes

We prioritized a gene by its pLI (cut-off set at ≥ 0.9) for frameshift, nonsense, and missense variants, which indicates intolerance to protein-truncating variation. Further, a Z-score cut-off was set by ≥ 3.09 as in the American College of Medical Genetics and Genomics guidelines recommended,^19^ indicating selection against missense variants in the respective gene.^21^ In addition, a missing entry in the genome aggregation database and our in-house database was considered.^20^ We additionally included a CADD score >20 for all variants. Variants with scores >20 are predicted to be among the 1% most deleterious possible substitutions in the human genome.^21^

### Statistical Analysis

Detailed statistical methods can be found in the Supplementary Material.

### Ethics Approval

This study was performed as per the standards of the Declaration of Helsinki 2013 and was approved by the local Ethics Committee of the Technical University of Munich (approval number: 521/16 S). Before inclusion, informed consent was obtained from each individual or their legal guardians.

## Results

### Study Population

The total study cohort comprised 101 index cases of VACTERL association and their 202 parents. The majority of the cases (62%) were male. In 1 case, a positive family history of VACTERL association was observed. One family was consanguineous. In 3 families, no precise clinical data were available (Table 1).

**Table 1:** Clinical characteristics of the 101 index cases

VACTERL, (V) vertebral defects, (A) anorectal malformations (ARM), (C) cardiac defect, (TE) tracheoesophageal fistula with or without esophageal atresia, (R) renal malformations, (L) limb defects.

### Assignment of Individuals

The recruited individuals were categorized as described in the Methods section to 1 of the following 2 groups based on the clinical phenotype of the index:

Group I (VACTERL group): There were a total of 89 of 101 (88%) affected index cases. The majority of the index cases (97%) had ARM, followed by renal abnormalities in 75% of the cases. Limb anomalies were the least common, accounting for 37% of the total cases.

Group II (VACTERL-like group): This group included 12 of 101 (12%) cases. The distribution of the affected organ systems in this group was relatively even. The most observed malformation was anorectal in 67% of the individuals, followed by other malformations in 55% and trachea esophageal malformations in 42% (Table 1).

Information of the clinical phenotype of all index cases is presented in Supplementary Table S1.

### Sample Preparation for Exome Sequencing

Owing to insufficient DNA quantity (*n* = 2), sample mismatch (*n* = 2), and sample contamination (1 parent in 1 family), 5 families were excluded (Figure 1). Exome sequencing was therefore performed in 96 index cases and their parents (trio exome sequencing).

### Identification of Disease-Causing Variants in Known Disease-Associated Genes

Disease-causing variants were identified in 5% (*n* = 5) of the index cases (Figure 1, Table 2). Of these, 4 individuals belonged to group I and the remaining 1 belonged to group II. Four of the 5 identified disease-causing variants were *de novo*; further, disease-causing variants inherited from 1 parent could not be identified.

**Table 2:** Phenotypic and genotypic details of index cases with disease-causing variants in known disease-associated genes

ID	Sex (determined genetically)	Gene (transcript)	Chromosomal position (hg19)	Nucleotide and amino acid change	Inheritance	Zygosity/heteroplasmy grade	gnomAD v.2.1.1 MAF^a^	Genetic diagnosis (MIM phenotype number)	Classification according to applied ACMG criteria/CNV score^b^	Individual ID in LOVD^c^
HN-F142-II-1	M	*KMT2D* (NM_003482.3)	chr12:49433509-49433509	c.8044C>T p.(Gln2682∗)	*de novo*	heterozygous	-	Kabuki syndrome 1 (147920)	Pathogenic PVS1, PS2, PM2, PP3	00435638
HN-F83-II-1	M	*KMT2D* (NM_003482.3)	chr12:49427394-49427395	c.11093dup p.(Phe3699Leufs∗14)	*de novo*	heterozygous	-	Kabuki syndrome 1 (147920)	Pathogenic PVS1, PS2, PM2	00435656
HN-F1249-II-1	F	*NSD1* (NM_022455.4)	chr5:176637649-176637650	c.2256_2257del p.(Pro753Lysfs∗11)	*de novo*	heterozygous	-	Sotos syndrome (117550)	Pathogenic PVS1, PM2, PP2	00435657
HN-F1406-II-1	M	Chr7p15.3-p21.2 del (-)	Approx. chr7:15405139-21985421	7736 kb deletion (including *TWIST1*)	*de novo*	heterozygous	-	Saethre-Chotzen syndrome (101400)	Pathogenic (1,45)	00435659
HN-F163-II-1	M	*MT-TL1* (NC_012920.1)	chrM:3243-3243	m.3243A>G	maternal	13%	-	MELAS syndrome (590050)	Pathogenic PS2_very strong, PS4_strong	00435658

ACMG, American College of Medical Genetics and Genomics; CNV, copy number variation; F, female; ID, identity; LOVD, Leiden Open Variation Database; M, male; MAF, minor allele frequency.

a https://gnomad.broadinstitute.org/

b Variant is classified as likely pathogenic/pathogenic as per ACMG and amendments ***18,19***.

c https://www.lovd.nl.

Kabuki syndrome was diagnosed genetically in 2 individuals. The first case (HN-F142-II-1; group II) had an aortic stenosis, patent ductus arteriosus, dysplastic mitral valve, atrioventricular septal defect II, renal hypoplasia, right pelvic kidney and a prostatic or bulbar urethra (Table 2). The second case (HN-F83-II-1; group I) had a tethered cord, ARM with rectoperineal fistula, hypoplastic left heart syndrome with mitral and aortic atresia, restrictive foramen ovale, coronary fistula, partial pulmonary vein misconnection of all left pulmonary veins, hydronephrosis on both sides, and a dilated right ureter.

The third case (HN-F1249-II-1; group I) had a Sotos syndrome and showed the clinical symptoms of scoliosis, bilateral hip dysplasia, bilateral clubfeet, syndactyly of the second and third toe, ARM with vestibular fistula, and a polycystic kidney on the right side.

The fourth case (HN-F1406-II-1; group I) had an approximately 8 Mb deletion and presented clinically with a Chiari malformation and tethered cord with caudal myelon adhesion, long-axis S-shaped scoliosis, ARM, fecal incontinence, right coronal synostosis, congenital vesicoureteral reflux, bilateral bow-leg drop feet, intellectual disability, postural instability, muscular hypotonia, congenital laryngomalacia, and choanal stenosis. The deletion included, among others, the disease-associated gene *TWIST1*, which has been associated with several allelic disorders, including Saethre-Chotzen syndrome.

The fifth case (HN-F163-II-1; group I) carried a known disease-causing variant in the mitochondrial gene *MT-TL1* with a heteroplasmy level of 13%. This individual showed an agenesis of the os coccygis, ARM with perineal fistula, esophageal atresia with tracheal fistula, retardation of bone age, and dental malformation, but no symptoms compatible with a mitochondriopathy. *MT-TL1* is associated with MELAS syndrome among others. The individual inherited this variant from his mother who had a heteroplasmy level of 3%.

### Identification of Non-synonymous *de Novo* and Rare Autosomal Recessive Variants in Unsolved Cases

**Figure 2:**
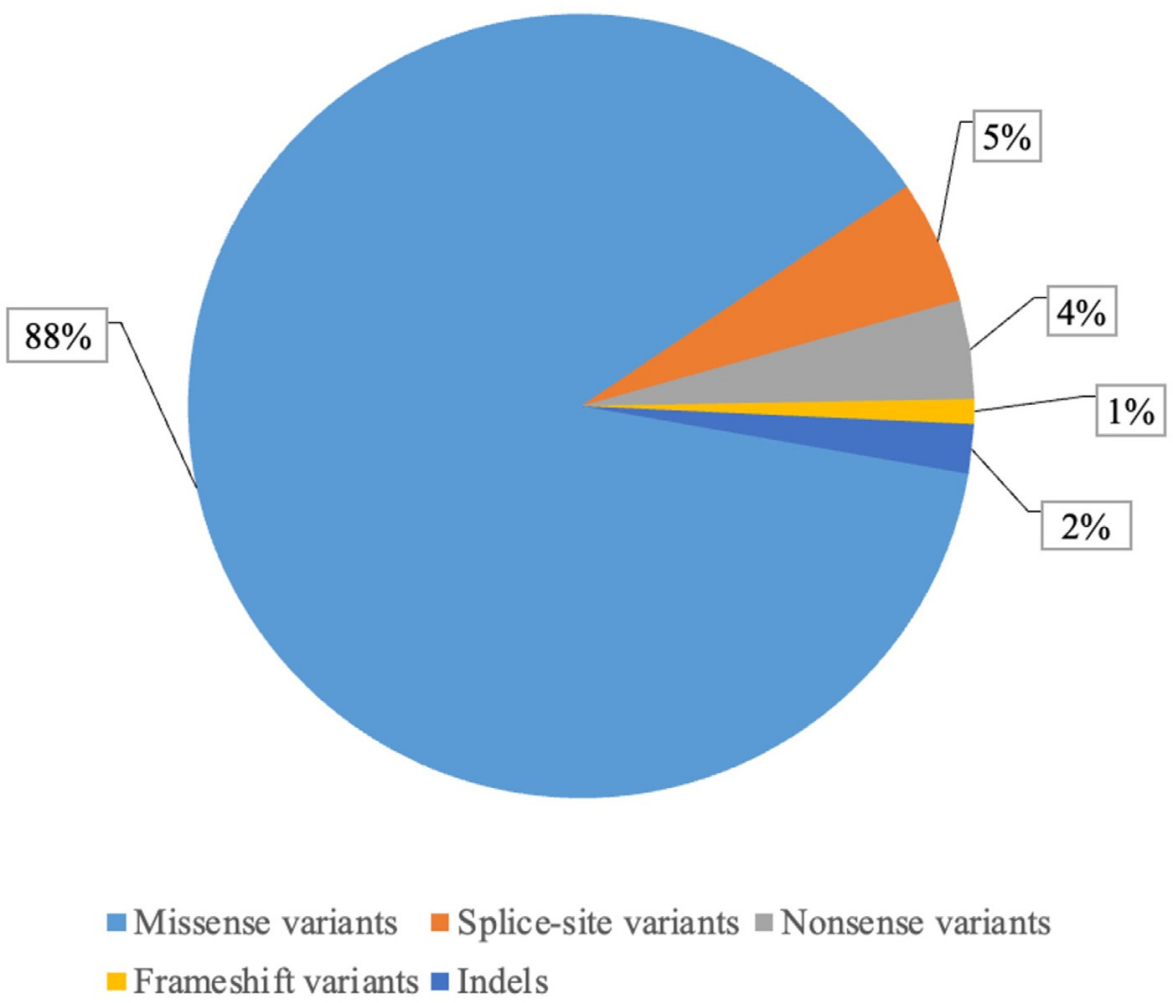
Distribution of 98 non-synonymous *de novo* variants in 97 different genes.

Among the remaining cases, 98 *de novo* variants were found in 97 different genes. Missense variants were the predominant type with up to 88%; splice site variants, 5%; nonsense variants, 4%; frame shift, 1%; and insertions and deletions, 2% (Figure 2, Supplementary Tables S2 and S3).

### Internal Prioritization of Candidate Genes

In total, 7 non-synonymous variants and 1 CNV in 6 candidate genes in 5 cases were prioritized. Five out of 8 (62.5%) variants were *de novo* compatible with an autosomal dominant inheritance (Supplementary Table S3).

With the autosomal recessive filter, we prioritized 1 individual who harbored 2 compound heterozygous variants and 1 individual who harbored a hemizygous variant (Supplementary Table S3).

### External Prioritization of Candidate Genes

Additional candidate genes were identified in 2 individuals who were coanalyzed in external studies: One case (HN-F162-II-1) had a heterozygous *de novo* missense variant NM_005324.5:c.361A>G; p.(Met121Val) in *H3F3B*. This individual presented with thoracic hemivertebra 10 and 11 on the left side, radial reduction malformation of the left forearm and hand, ARM without fistula, hypoplastic left heart syndrome, atrioventricular septal defect, and horseshoe kidney. This individual was also included in another project during this study and is already part of the publication of Bryant et al. 2020.^22^

The case study involving HN-F136-II-1 and its variant NM_006885.3:c.6377C>T; p.(Ala2126Val) in *ZFHX3* was recently published.^23^ This individual had ARM with a rectovesical fistula, esophageal atresia, an atrioventricular septal defect II, and a renal agenesis on the right side.

### Analysis of a Gene and Pathway Burden Test

A total of 96 VACTERL cases (60 male, 36 female) and their parents were analyzed by gene and pathway burden test. Of the internal controls, 134 were male and 125 were female.

**Figure 3:**
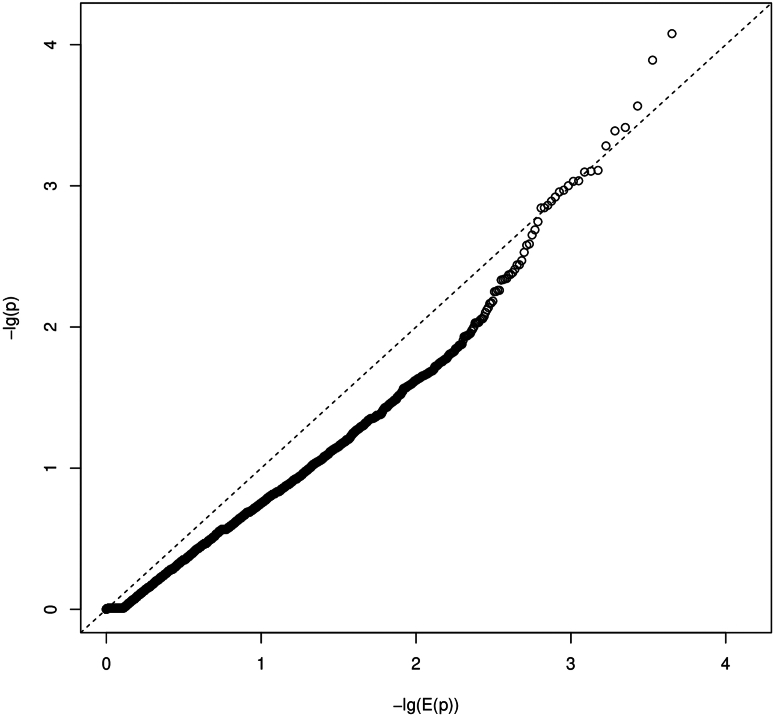
Quantile-quantile plot (QQ-plot) for gene burden. The plot shows the distribution of the sorted *P*-values from data analysis against the expected uniform distribution. −lg(E(*P*)): expected −log_10_*P*-values; −lg(*P*): observed −log_10_*P*-values.

**Figure 4:**
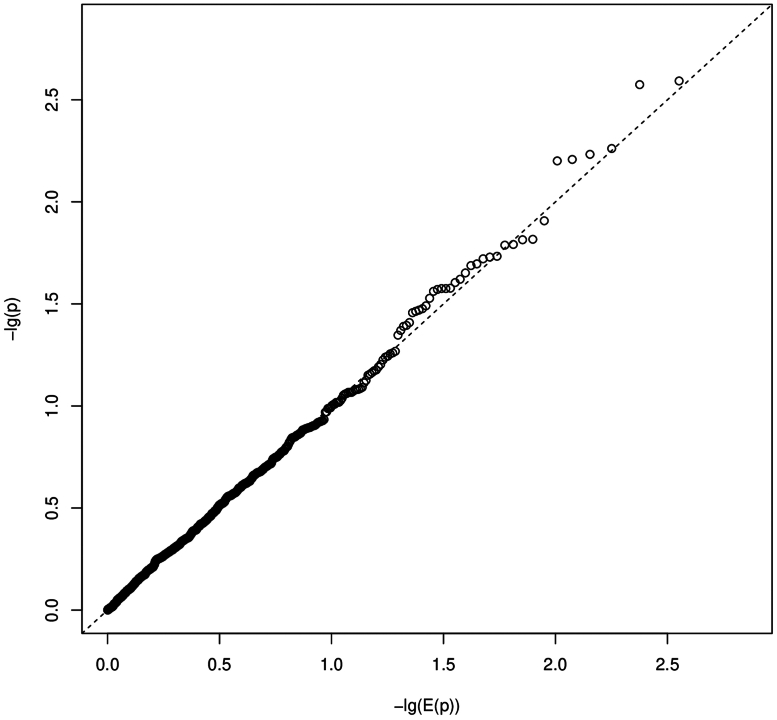
Quantile-quantile plot (QQ-plot) for pathway burden. The plot shows the distribution of the sorted *P*-values from data analysis against the expected uniform distribution. −lg(E(*P*)): expected −log_10_*P*-values; −lg(*P*): observed −log_10_*P*-values.

We obtained burden measures for 13,480 genes and 712 pathways. Regression models with random effects had rank deficient variance-covariance matrices for the random effects and were therefore excluded from further analysis. From the models with only fixed effects, we estimated a number of 339 independent pathway burden hypotheses. Therefore, multiple-testing corrected significance threshold was approximately *P* ≤ 3.7E-6 for gene burden tests and *P* ≤ 1.5E-4 for pathway burden tests. None of the *P*-value showed statistical significance. The smallest *P*-value was observed for the gene *HECTD1* (*P* = 2.9E-5; Supplementary Material) and the pathway "WP_FBXL10_ENHANCEMENT_OF_MAPERK_SIGNALING_IN_DIFFUSE_LARGE_BCELL_LYMPHOMA" (*P* = 0.0007). The quantile-quantile plots did not show a strong deviation of *P*-values from expected random chance *P*-values for most of the tests (Figure 3, Figure 4). The estimated inflation of *P*-values (Lambda values) for the case-control burden tests were 0.42 and 1.03 for gene-level and pathway-level tests, respectively.

**Figure 5:**
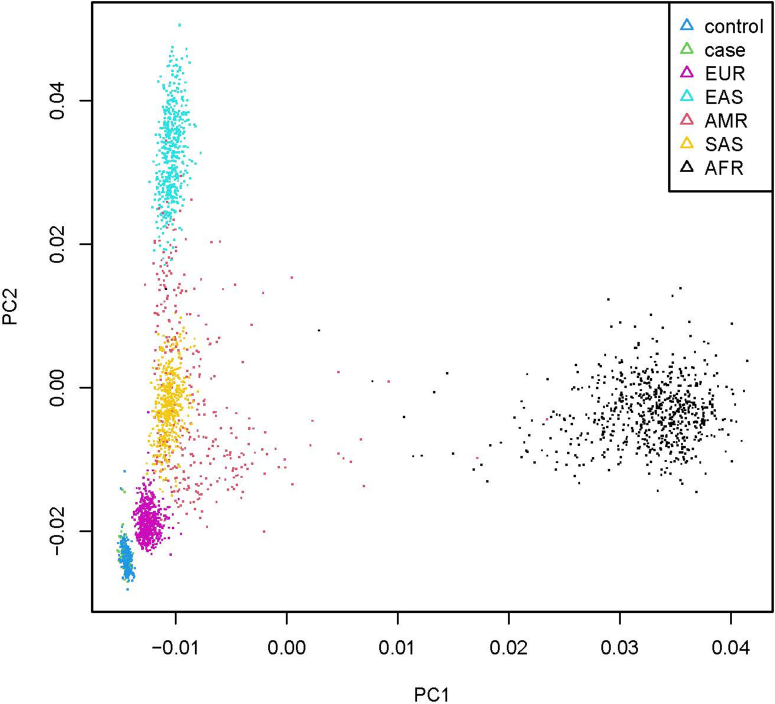
PCA plot of VACTERL individuals and control individuals compared with the 1000 genomes sample.There is a single cluster for combined cases and controls, which maps close to the EUR cluster of 1000 genomes samples. PCA, principal component analysis.

We additionally compared the unrelated VACTERL individuals (*n* = 96) and unrelated controls (*n* = 252, 7 controls were removed because of relatedness) with the 1000 genomes sample [*n* (AFR) = 661, *n* (AMR) = 347, *n* (EAS) = 504, *n* (EUR) = 503, and *n* (SAS) = 489] (The 1000 Genomes Project Consortium, 2015). After filtering the variants, we obtained principal components based on 1300 variants. Visual inspection of the first 2 principal components showed a single cluster for combined cases and controls, which mapped close to the EUR cluster of 1000 genome samples (principal component analysis; Figure 5).

**Figure 6:**
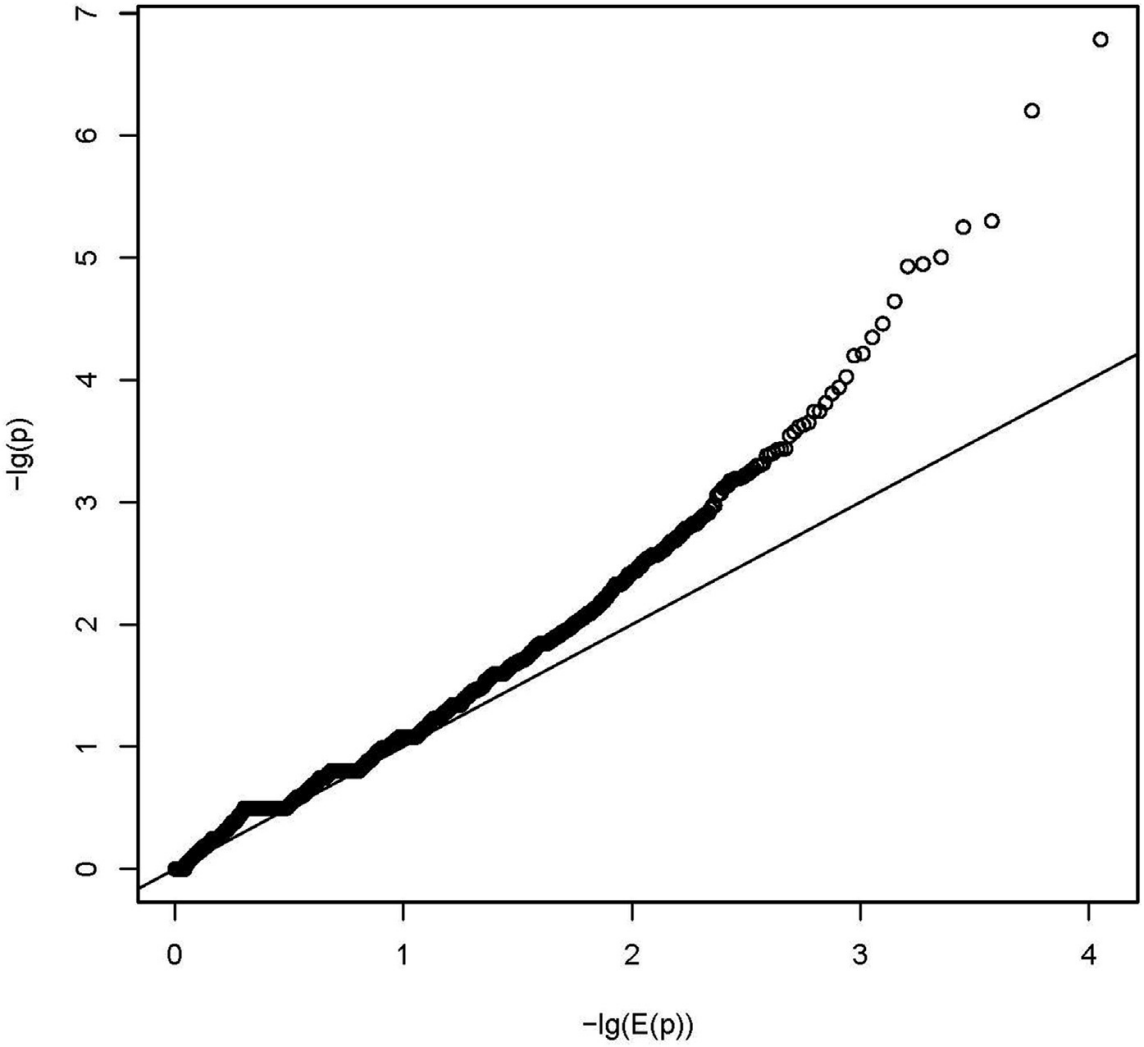
Quantile-quantile plot (QQ-plot) for gene burden on FBAT analysis. The plot shows the distribution of the sorted *P*-values from data analysis against the expected uniform distribution. −lg(E(*P*)): expected −log_10_*P*-values; −lg(*P*): observed −log_10_*P*-values.

**Figure 7:**
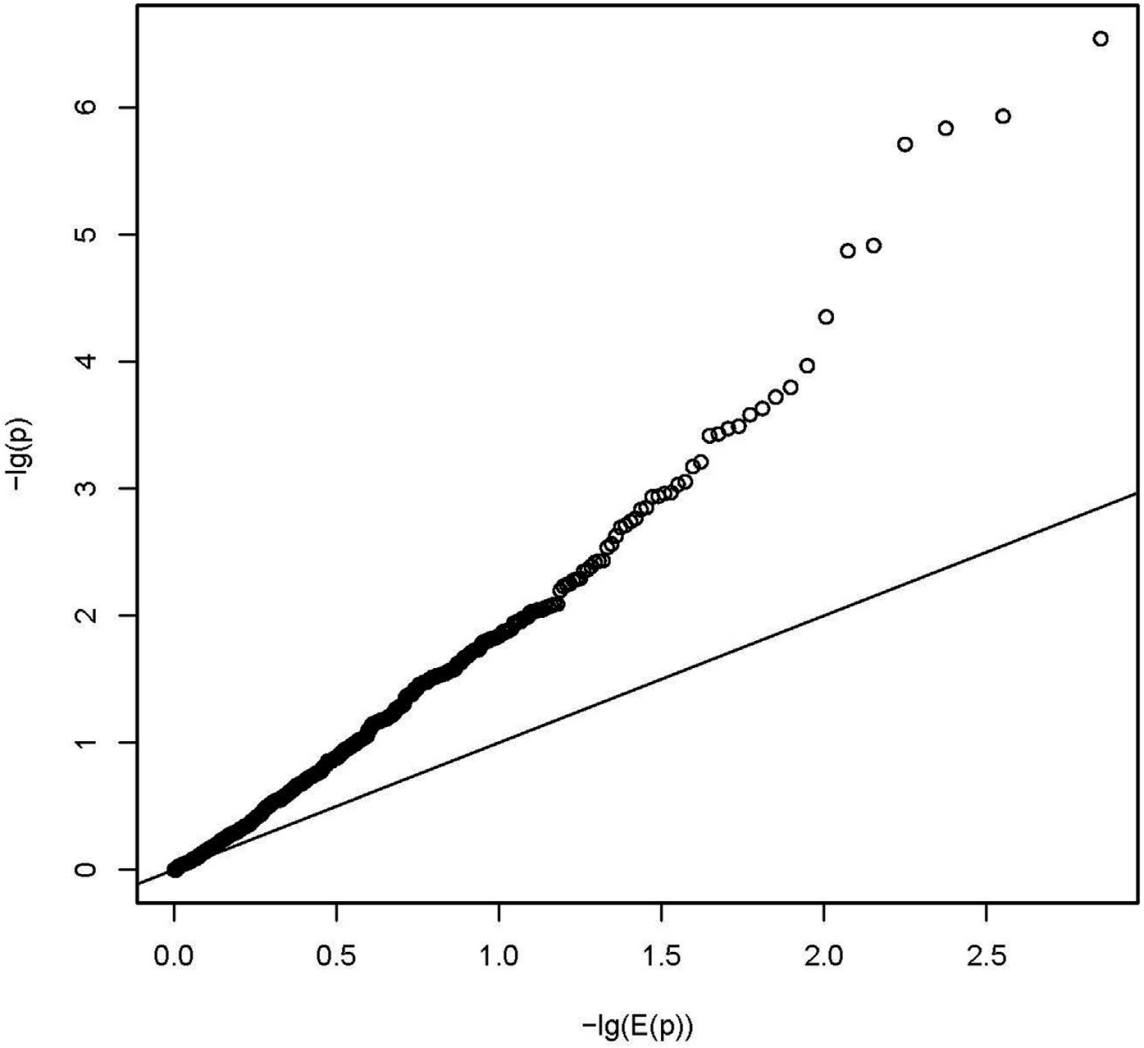
Quantile-quantile plot (QQ-plot) for pathway burden on FBAT analysis. The plot shows the distribution of the sorted *P*-values from data analysis against the expected uniform distribution. −lg(E(*P*)): expected −log_10_*P*-values; −lg(*P*): observed −log_10_*P*-values.

**Figure 8:**
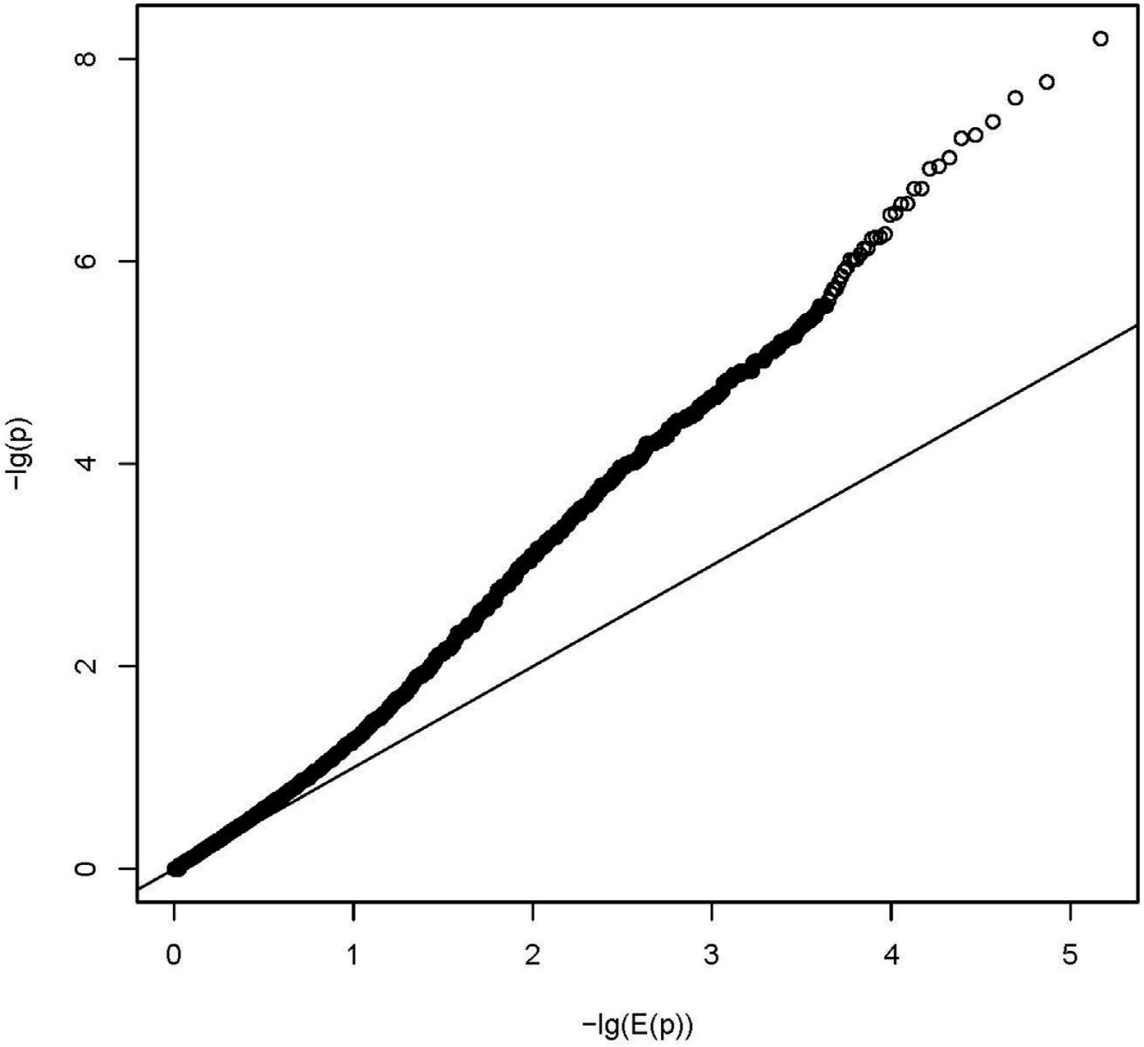
Quantile-quantile plot (QQ-plot) summary on FBAT analysis. The plot shows the distribution of the sorted *P*-values from data analysis against the expected uniform distribution. −lg(E(*P*)): expected −log_10_*P*-values; −lg(*P*): observed −log_10_*P*-values.

In addition to case-control burden association tests, we performed FBAT on the nuclear families from our index cases.^24^ We tested 147,837 of initial 473,238 single variants (no functional constraints), 11,287 genes, and 709 pathways (function constraint on variants). We adjusted for multiple testing (Bonferroni) for 147,837 variants, 11,287 gene-level tests, and 339 independent pathway-level tests (as for the burden analysis), respectively. We observed 14 significant variants (lead variant chr19:14877817 T > A, *P* = 2.26E-09; Supplementary Table S4), 2 significant genes (*LOC645752*, *P* = 1.64E-07, and *ZNF417*, *P* = 6.25E-07; Supplementary Table S5), and 8 significant pathways (lead pathway WP_EBOLA_VIRUS_INFECTION_IN_HOST, *P* = 2.88E-07, cell cycle and NOTCH signaling pathway; Supplementary Table S6). The inflation of the test statistics were 1.27, 2.15, and 2.38, respectively (Figure 6, Figure 7, Figure 8).

## Discussion

Despite immense recent technological advancements, the etiology of VACTERL association remains mostly unknown. Underlying reasons include the phenotypic and causal heterogeneity, the typically sporadic nature of the disease, and the large number of overlapping syndromes.

The present study highlights the variable diagnostic yield of molecular genetic analysis in individuals associated with VACTERL association. Disease-causing variants were identified in only 5% of the index cases indicating a low diagnostic yield in this cohort but could identify several candidate genes. The yield of 5% indicates a more complex genetic architecture compared with other kidney disorders, such as nephronophthisis with a detection rate of 63% or tubulopathies with a detection rate between 26% and 50%.^25^, ^26^, ^27^ The observed low rate here suggests that this syndromic condition may involve a larger number of genes, unique genetic mechanisms, or a high degree of phenotypic variability, making genetic diagnosis more challenging. The genetic landscape of each condition is shaped by factors such as the number of implicated genes, the diversity of variants, and the accessibility of comprehensive genetic testing methods. The presence of overlapping genetic disorders such as Saethre-Chotzen syndrome or Kabuki syndrome in VACTERL association pose challenge to the accurate diagnosis of the VACTERL association and complicates diagnostic accuracy.

One recent research group was able to make a diagnosis using exome sequencing in 16% (11/67) of the individuals with isolated esophageal atresia or tracheoesophageal fistula (EA/TEF).^28^ In another study, molecular diagnosis could be made in 27% (19/71) of the individuals presenting with a VACTERL or VACTERL-like phenotype. The solved cases harbored disease-causing variants in disease-associated genes such as *ADNP*, *BBS1*, *FGFR3*, *KMT2D*, *LRP2*, *NIPBL*, and *SALL1,* known for a variety of syndromes.^29^ Kolvenbach et al. 2021 were able to identify in 6 of 21 (29%) cases, potentially disease-causing variants in the genes *B9D1*, *FREM1*, *ZNF157*, *SP8*, *ACOT9*, *B9D1, SP8, ACOT9,* and *TTLL11*,^3^ but only 2 of these genes (*B9D1*, *FREM1*) have been described as disease-associated in the literature so far. In contrast to those findings, and despite careful clinical selection, we only diagnosed a monogenic disease in 5% (5/96) of the cases. The discrepancies in the first study on individuals with esophageal atresia or tracheoesophageal fistula may be due to the isolated phenotype in these individuals and thus a different, more often monogenic genesis. In the second study mentioned above, significantly more individuals with a complex clinical phenotype and likely a different—monogenic inherited—disease entity may have been included, which is probably the reason for the higher diagnostic yield. When only the individuals with disease-causing variants in known disease-associated genes were considered in Kolvenbach’s study, the proportion of solved individuals decreased to 10%, which is comparable with ours.

Interestingly, overlapping deletions have been previously described in the literature in several cases with a phenotype similar to our fourth case (HN-F1406-II-1).^30^^,^^31^ Carriers of loss-of-function variants in *TWIST1* exhibit a craniofacial malformation syndrome with abnormalities of the facial skull, craniosynostosis, syndactyly, and incomplete duplication of the great toe, among others.^32^ In affected individuals with major deletions involving other genes besides *TWIST1*, developmental delay and variable organ malformations (including anal stenosis and another ARM) have been described.^30^ Taken together, this suggests that individuals with a Saethre-Chotzen syndrome may be misdiagnosed as individuals with a VACTERL association (https://www.ncbi.nlm.nih.gov/books/NBK1204/table/bgs.T.disorders_to_consider_in_the_diffe/).

Our data and those of Belanger Deloge et al. 2022 suggest that molecular genetic analyses designed to identify monogenic etiologies may have a lower diagnostic yield in cases who initially meet the criteria for VACTERL association.^29^ VACTERL has not yet been satisfactorily explained in terms of pathogenesis. Few familial cases have been identified, and external factors such as maternal diabetes, assisted reproductive techniques, maternal pregestational overweight and obesity, as well as maternal smoking seem to play an influencing role.^4^ Disease-causing variants in single genes have been found in a number of syndromes with 1 or more malformations seen in individuals with the VACTERL association; however, these syndromes additionally have other features that distinguish them from VACTERL association.^33^ Sotos and Kabuki syndrome are 2 of those syndromes which have actually highly recognizable phenotypes that could be differentially diagnosed by a clinical geneticist.

Abnormal mitochondrial function has rarely been described in individuals with VACTERL association.^34^^,^^35^ To our knowledge, however, our case appears to be the second case described in the literature in which the np 3243 variant occurs. Interestingly, it did not show the typical symptoms of MELAS.^36^ Damian et al.,^34^ presented a female individual with VACTERL association with the np 3243 variant. The girl was born with multiple malformations of the hands, face, ribs, multiple cervical and thoracic vertebral anomalies, congenital anomalies of the kidney and urinary tract, an ARM with rectovestibular fistula, moderate global cardiac dilatation, and died at the age of 1 month.^34^ To date, 7 individuals, ours included, have been found to have both VACTERL association and mitochondrial dysfunction.^35^ Nevertheless, it cannot be ruled out with certainty that this variant is a secondary finding and not the actual cause because our case did not show any symptoms compatible with MELAS. It should therefore be carefully considered whether mitochondrial work-up should be included in the molecular genetic testing.

Within the present study, 5 candidate genes were identified. One of them was *GCN1L1*, which has previously been prioritized as one of the tumor-associated genes in individuals with renal carcinoma, suggesting that *GCN1L1* may participate in renal carcinogenesis via Wnt/β-catenin signaling.^37^ The importance of the Wnt pathway in kidney development in humans is well-known and is already confirmed by mouse models.^38^ Our index case, HN-F151-II-1 had a vesicoureteral reflux, renal hypoplasia, and bilateral heterozygous variants in *GCN1L1*, implying that this gene may also play an important role in kidney development.

Individual HN-F158-II-1 harbored a heterozygous missense variant in *GREB1L* with a Z-score >5 and with an LOEF of 0.07. This variant was described twice in ClinVar as likely pathogenic and is also a known disease-associated gene for renal hypodysplasia or aplasia 3. There are 2 other affected individuals and 1 affected mother described in De Tomasi et al., 2017, with the clinical picture of a vesicoureteral reflux and a heterozygous variant in the *GREB1L*.^39^ Our index case harbored the heterozygous missense variant NM_001142966.2:c.2252G>A, inherited from his unaffected mother. Intrafamilial phenotypic variability focused on renal phenotype up to incomplete penetrance, is already described for individuals with disease-causing variants in this gene and is therefore not an exclusion criterion for the identified variant.^39^ Nevertheless, it does not explain the syndromal appearance of our index case, which could be caused by a multifactorial effect or possible polygenic inheritance.

Finally, to further elucidate the VACTERL conundrum, we evaluated the burden of putatively damaging rare variants in individuals with VACTERL association from exome sequencing in affected individuals, unaffected parents, and controls. Including relatedness from the pedigree structure likely leads to a model overfitting, which could lead to an inflation of *P*-values for the burden tests, and therefore result in detection of pathways just by chance. Dropping the random effect from the models, the quantile-quantile plots did not show strong deviation of burden *P*-values for the genes and pathways from chance *p*-values. Thus, neither relatively strong bias (e.g., population substructure, unobserved confounders) nor relatively strong polygenic effects could be suggested. No *P*-value was significant after multiple testing. These tests depend on the sample size, heritability, linkage disequilibrium, and the number of causal variants.^40^ In addition, the principal component analysis plot was in accordance with the origin of the included individuals, both in terms of the affected individuals and the controls. Further FBAT at single variant, gene, and pathway levels showed isolated significant results on loci not associated with VACTERL association so far but will be subject of further examinations. It should be mentioned here that the FBAT-significant gene *ZNF417* is a potential VACTERL candidate gene, encoding for a ubiquitously expressed zinc finger protein, which is part of the well-known Krüppel-associated box zinc finger protein family regulating transcription (https://www.gtexportal.org/home/gene/ZNF107).^41^ The same applies to the observed FBAT-significant pathways (e.g., cell cycle and NOTCH signaling pathway, Supplementary Table S6). These are known to be involved in kidney development, which therefore should be further examined.^42^^,^^43^

There are several limitations of this study. Although this is the largest study conducted on individuals with VACTERL association, the sample size may have been too small and therefore, probably not significant enough to reveal substantial pathophysiologic links. Published burden analysis includes sample sizes 1 to 2 orders of magnitude larger than our data set. Therefore, we analyzed the burden specifically of whole pathways, which reduced the multiple testing problem and increased the number of observations per test. However, when genes in a pathway have opposite effects (i.e., some being detrimental and some protective), this approach would lack power because the opposite effects would cancel each other in the burden effect for the pathway. Variance-components methods such as sequence kernel association test would be an alternative in that case; however, it would increase the multiple testing problem when performed in addition to the burden tests. Sequence kernel association test can (if the cohort is moderate or large) be a powerful approach to identify new candidate genes, or to design new genome sequencing or exome sequencing studies in a very short time and without any previous functional information.^44^

In our study, control individuals were persons suffering from other diseases, as well as healthy relatives. Therefore, significant sequence kernel association test results may be difficult to interpret, because a *P*-value may be driven by causal variants of these other diseases and not by variants causal for the disease of interest. This also applies to the burden tests; however,the direction of the effect is easier to interpret. The analysis did not apply any allele frequency cut-off. Functional variants have a broad spectrum of allele frequencies ranging from rare to common, which is a realistic model for complex traits.^45^

When analyzing larger data sets in the future, relatedness structure and additional covariates such as age are likely to be explanatory confounders in fitting the model and may have to be included. It is also recommended to use population-based controls instead of cases of other diseases.

Some further limitations arise from molecular genetic analysis. For example, repeat expansions and deep intronic variants should be mentioned, both of which are not detectable with exome sequencing. In addition, a unified phenotype for VACTERL cases with presumed same malformations is difficult to define. Further, there may be a bias in recruitment because our cohort included individuals from the self-help organizations for individuals with congenital ARMs.

We are aware that for the calculation of a burden test, the low number of cases is one of the limiting factors besides the cofounder variants, technical artefacts, and missing calls (multisampling calling). Burden tests have a crucial role in the presence of both trait-increasing and trait-decreasing variants, or in the presence of a small fraction of causal variants and has been in mind as potential limitation.

Combining insights from biological models with the newly available genomic technologies may provide more answers to causal mechanisms in the near future. With further targeted research, these answers may then address an even more important question: How can the health of affected individuals be improved?

If the clinical presentation meets the traditional diagnostic criteria of VACTERL association (at least 3 characteristic features) without additional syndromic features or intellectual disability, diagnosis is made on clinical assessment alone. In such scenarios, the reduced probability of identifying a causative variant may limit the power of exome sequencing.^46^^,^^47^ Conversely, in individuals with atypical features such as neurodevelopmental delay or other syndromic manifestations that differ from the traditional VACTERL profile, exome sequencing has been valuable. It facilitates the identification of potential underlying syndromes that mimic VACTERL features, which can have a significant impact on both the management and prognosis of the individual.^3^^,^^46^ The decision to perform exome sequencing should be the result of a comprehensive clinical assessment in which the specific characteristics and manifestations of each individual are taken into account. It may be more appropriate to focus on clinical management and routine follow-up when traditional diagnostic criteria are clearly met, and no additional symptoms are evident.

## Conclusion

The current literature suggests that the underlying cause of VACTERL association is multifactorial. Our study confirms this, because disease-causing variants in known disease-associated genes were found in only 5 out of 96 individuals. Nonetheless, additional burden analyses identified promising candidate genes and pathways, possibly involved in the development of VACTERL association. However, it is obvious that other genetic or non-genetic causes, which cannot be captured with short-read-based exome sequencing, are likely involved in VACTERL. Future research should therefore rely on a broader scope of techniques, including genome/RNA sequencing, long-range sequencing, chromosome conformation analysis, and epigenetics to identify novel disease mechanisms and to better understand the pathophysiology of VACTERL.

## Disclosure

All the authors declared no competing interests.

## Supplementary Material

Supplementary File (PDF) **Supplementary Material. Supplementary References. STROBE Checklist. Web Resources**.

Supplementary File (Excel) **Table S1.** Phenotypic characteristics of 101 affected index cases and their unaffected parents. **Table S2.** Non-synonymous *de-novo* variants identified in the unsolved index cases. **Table S3.** Selection of highly promising candidate genes and their variants identified in the unsolved cases. **Table S4.** FBAT on single variant level. **Table S5.** FBAT on gene level. **Table S6.** FBAT on pathway level. **STROBE Checklist**

## Footnotes

Supplementary File (PDF)

**Supplementary Material.**

**Supplementary References.**

**STROBE Checklist.**

**Web Resources.**

Supplementary File (Excel)
**Table S1.** Phenotypic characteristics of 101 affected index cases and their unaffected parents. **Table S2.** Non-synonymous *de-novo* variants identified in the unsolved index cases. **Table S3.** Selection of highly promising candidate genes and their variants identified in the unsolved cases. **Table S4.** FBAT on single variant level. **Table S5.** FBAT on gene level. **Table S6.** FBAT on pathway level.
