# Supplementary material for: Trio Exome Sequencing in VACTERL Association
Source: Kidney Int Rep. 2024 Dec 9;10(3):877–91. doi: 10.1016/j.ekir.2024.12.006 (PMC11993224; doi:10.1016/j.ekir.2024.12.006)
Supplement: Supplementary File (PDF) — Supplementary Material. Supplementary References. STROBE Checklist. Web Resources. [file mmc1.pdf]

Rare disease-causing variants in VATER/VACTERL association

Supplementary Material

Statistical analysis

Data preparations

All data preparation and analysis were performed in R (4.1.1). Burden of ES variant alleles per individual and gene was extracted from our in-house NGS variant and annotation database (EvaDB) (R packages RMariaDB_1.2.2 and lubridate_1.9.0). The test was performed on our index cases, their parents, and internal controls to improve statistical power (individuals not affected by a developmental disorder, heart disease, mitochondrial diseases, or myocardial infarction). Variant calls originated from single sample variant calling. Variants were filtered based on mapqual greater/equal 50 and coverage greater/equal 15. Only missense, nonsense, stoploss, splice, frameshift, and indel variants were considered. To further increase power, burden by genes was further collapsed into burden by pathways. Pathways were downloaded from www.gsea-msigdb.org (wikipathways.v2022.1, accessed on 3rd Feb 2023).

Statistical analysis

We fitted a model with a logistic regression framework, with affection status being the response and either a) burden and sex being fixed effects (R function glm), or b) burden and sex being fixed effects and additionally relatedness (inferred from the pedigree) being a random effect (R packages pedigreemm_0.3-3 and lme4_1.1-31).[S1]

We assessed the compatibility between our data and a model where the burden has no effect (null hypothesis, as evaluated with significance of *p*-values from Wald statistics of the fitted model). The *p*-value threshold for the burden effect from a fitted model was preset to 0.05 to

meet an assumed type-I-error rate of 0.05. However, we would expect a proportion of 0.05 of all our tests to be detected as significant by chance. To avoid this, we applied Bonferroni multiple testing correction (i.e., *p*-values had to be less/equal than the expected type-I-error divided by number of tests to be significant). We tested both variant burden by gene and variant burden further collapsed into pathways (and applied Bonferroni correction for both testing schemes separately). As pathways share genes, the respective burden measures and *p*-values are not independent, thus they are redundant to some degree. As a consequence, correction by the number of pathways would be too conservative and lead to a loss of statistical power to detect a true (i.e. non-zero) effect of the burden. Therefore, we estimated the number of independent tests: We permuted the phenotype in our data 10,000 times randomly, each time calculating *p*-values for all pathways' burden and calculated a correlation matrix for the pathways' burden effects (R function cor, method "spearman"). From that correlation matrix, we estimated the number of independent burden tests (R package poolr_1.1-1, function meff with method "galwey") for the Bonferroni correction.[S2] We additionally created a quartile-quartile plot (QQ-plot) by plotting the negative lg of the sorted observed burden *p*-values against the negative lg of sorted random *p*-values, to compare the shapes of distribution.

We further compared our index individuals and our control samples to 1000 genomes samples (phase 3, v5b.20230502) (The 1000 Genomes Project Consortium, 2015): We merged all variant calls (DP >= 15, MQ >= 50, QD >= 30, no function restraints) with 1000 genomes genotypes, and kept variants with MAF >= 1% and call rate >= 95% (using PLINK v1.07).[S3] We calculated the first 10 principal components using PLINK v1.90b3.35.[S4]

To test for association and linkage simultaneously, we applied FBAT (v.2.0.8) to our VACTERL patients and their nuclear families.[S5, S6] We tested on a single variant (DP >= 15, MQ >= 50) without function constraints, additive genetic effects, constraint on at least 10 informative families (standard setting), and VACTERL population prevalence of 0.0001. We also tested on gene and pathway level (command "fbat -v0") using the variants that were used

for the burden test before (functional constraint on deleterious variants), an additive genetic effect model, and at least 1 informative family per variant (standard settings). FBAT software removes Mendelian errors from the data and therefore cannot include *de novo* variants into the analysis.

Supplementary References

S1. Vazquez AI, Bates DM, Rosa GJ, *et al.* Technical note: an R package for fitting generalized linear mixed models in animal breeding. *J Anim Sci* 2010; **88:** 497-504.

S2. Cinar O, Viechtbauer W. The poolr Package for Combining Independent and Dependent p Values. *Journal of Statistical Software* 2022; **101:** 1 - 42.

S3. Purcell S, Neale B, Todd-Brown K, *et al.* PLINK: a tool set for whole-genome association and population-based linkage analyses. *Am J Hum Genet* 2007; **81:** 559-575.

S4. Chang CC, Chow CC, Tellier LC, *et al.* Second-generation PLINK: rising to the challenge of larger and richer datasets. *Gigascience* 2015; **4:** 7.

S5. Rabinowitz D, Laird N. A unified approach to adjusting association tests for population admixture with arbitrary pedigree structure and arbitrary missing marker information. *Hum Hered* 2000; **50:** 211-223.

S6. Zhou JJ, Yip WK, Cho MH, *et al.* A comparative analysis of family-based and population-based association tests using whole genome sequence data. *BMC Proc* 2014; **8:** S33.

STROBE Statement—Checklist of items that should be included in reports of ***cohort studies***

	Item No	Recommendation	Page No
**Title and abstract**	1	(*a*) Indicate the study's design with a commonly used term in the title or the abstract<br>(*b*) Provide in the abstract an informative and balanced summary of what was done and what was found	1<br>4
**Introduction**			
Background/rationale	2	Explain the scientific background and rationale for the investigation being reported	5-6
Objectives	3	State specific objectives, including any prespecified hypotheses	6
**Methods**			
Study design	4	Present key elements of study design early in the paper	7
Setting	5	Describe the setting, locations, and relevant dates, including periods of recruitment, exposure, follow-up, and data collection	7
Participants	6	(*a*) Give the eligibility criteria, and the sources and methods of selection of participants. Describe methods of follow-up<br>(*b*) For matched studies, give matching criteria and number of exposed and unexposed	7
Variables	7	Clearly define all outcomes, exposures, predictors, potential confounders, and effect modifiers. Give diagnostic criteria, if applicable	7
Data sources/ measurement	8*	For each variable of interest, give sources of data and details of methods of assessment (measurement). Describe comparability of assessment methods if there is more than one group	7-9
Bias	9	Describe any efforts to address potential sources of bias	-
Study size	10	Explain how the study size was arrived at	7
Quantitative variables	11	Explain how quantitative variables were handled in the analyses. If applicable, describe which groupings were chosen and why	-
Statistical methods	12	(*a*) Describe all statistical methods, including those used to control for confounding<br>(*b*) Describe any methods used to examine subgroups and interactions<br>(*c*) Explain how missing data were addressed<br>(*d*) If applicable, explain how loss to follow-up was addressed<br>(*e*) Describe any sensitivity analyses	9
**Results**			
Participants	13*	(a) Report numbers of individuals at each stage of study—eg numbers potentially eligible, examined for eligibility, confirmed eligible, included in the study, completing follow-up, and analysed<br>(b) Give reasons for non-participation at each stage<br>(c) Consider use of a flow diagram	10
Descriptive data	14*	(a) Give characteristics of study participants (eg demographic, clinical, social) and information on exposures and potential confounders<br>(b) Indicate number of participants with missing data for each variable of interest<br>(c) Summarise follow-up time (eg, average and total amount)	10-13
Outcome data	15*	Report numbers of outcome events or summary measures over time	-

Main results	16	(*a*) Give unadjusted estimates and, if applicable, confounder-adjusted estimates and their precision (eg, 95% confidence interval). Make clear which confounders were adjusted for and why they were included<br>(*b*) Report category boundaries when continuous variables were categorized<br>(*c*) If relevant, consider translating estimates of relative risk into absolute risk for a meaningful time period	10-14
Other analyses	17	Report other analyses done—eg analyses of subgroups and interactions, and sensitivity analyses	13-14
**Discussion**			
Key results	18	Summarise key results with reference to study objectives	15
Limitations	19	Discuss limitations of the study, taking into account sources of potential bias or imprecision. Discuss both direction and magnitude of any potential bias	19-20
Interpretation	20	Give a cautious overall interpretation of results considering objectives, limitations, multiplicity of analyses, results from similar studies, and other relevant evidence	15-21
Generalisability	21	Discuss the generalisability (external validity) of the study results	21-22
**Other information**			
Funding	22	Give the source of funding and the role of the funders for the present study and, if applicable, for the original study on which the present article is based	23

*Give information separately for exposed and unexposed groups.

Note: An Explanation and Elaboration article discusses each checklist item and gives methodological background and published examples of transparent reporting. The STROBE checklist is best used in conjunction with this article (freely available on the Web sites of PLoS Medicine at http://www.plosmedicine.org/, Annals of Internal Medicine at http://www.annals.org/, and Epidemiology at http://www.epidem.com/). Information on the STROBE Initiative is available at http://www.strobe-statement.org.

SUPPLEMENTARY MATERIAL

WEB RESOURCES

1. ClinVar: https://www.ncbi.nlm.nih.gov/clinvar/
2. Database of Genomic Variants (DGV): http://dgv.tcag.ca/dgv/app/home
3. dbSNP, http://www.ncbi.nlm.nih.gov/SNP/
4. DECIPHER: https://decipher.sanger.ac.uk/
5. Human Gene Mutation Database (HGMD® Professional): http://www.hgmd.cf.ac.uk
6. Genome Aggregation Database (gnomAD): https://gnomad.broadinstitute.org/
7. Integrative Genomics Viewer (IGV): https://software.broadinstitute.org/software/igv/
8. Leiden Open Variation Database: https://www.lovd.nl
9. Online Mendelian Inheritance in Man (OMIM). Johns Hopkins University: http://www.omim.org
10. PolyPhen2, http://genetics.bwh.harvard.edu/pph2/
11. SankeyMATIC: http://sankeymatic.com/build/
12. UniProt, https://www.uniprot.org/
13. Wikipathways: www.gsea-msigdb.org
